# The prognostic value of Forkhead box P3 regulatory T cells in biliary tract cancer: A systematic review and meta-analysis

**DOI:** 10.1097/MD.0000000000036608

**Published:** 2023-12-15

**Authors:** Pengcheng Cai, Zhongli Wu, Xingjian Yang, Na Wang, Yong Yang

**Affiliations:** a Department of General Surgery, the First People’s Hospital of Shuangliu District, Chengdu, Sichuan Province, China.

**Keywords:** biliary tract cancer, FoxP3^+^, meta-analysis, prognosis, regulatory T cells

## Abstract

**Background:**

This study aimed to explore the value of tumor-infiltrating Forkhead box P3(FoxP3^+^) regulatory T cells (Tregs) in evaluating the prognosis of biliary tract cancer.

**Methods:**

Four electronic databases were searched using 2 computers: PubMed, Embase, Web of Science, and Cochrane Library. The vocabulary and syntax were adapted according to the database. Two researchers independently selected the studies, collected information, and assessed the risk of bias. The Meta-analysis was performed using STATA 17.0, and HR and its corresponding 95% CI were used to evaluate the correlation between FoxP3^+^ Tregs and the overall survival of patients with biliary tract cancer. In addition, the quality of the included studies was evaluated.

**Results:**

Ten articles were included in this study. The results of the meta-analysis showed that patients with high FoxP3^+^ Tregs infiltration had worse overall survival (OS) (HR = 1.34,95% CI 1.16 to 1.71; *P* < .001). Subgroup analysis of gallbladder carcinoma and cholangiocarcinoma showed that the high infiltration of FoxP3^+^ Tregs was significantly correlated with the OS of the former (HR = 1.55,95% CI 1.11 to 2.00; *P* < .001), but not with the OS of the latter (HR = 1.00,95% CI 0.62 to 1.38; *P* > .05).

**Conclusions:**

Our meta-analysis reveals that high infiltration of FoxP3 + Tregs is significantly associated with reduced overall survival in gallbladder carcinoma, endorsing their use as a prognostic biomarker for this subtype. In contrast, no significant prognostic correlation was identified for FoxP3^+^ Tregs in cholangiocarcinoma, indicating the need for subtype-specific evaluation of their prognostic relevance in biliary tract cancers.

## 1. Introduction

Biliary tract cancer, encompassing gallbladder carcinoma, extrahepatic cholangiocarcinoma, and intrahepatic cholangiocarcinoma, is an infrequent malignancy affecting the digestive tract. This type of cancer is associated with a grim prognosis. The poor prognosis of this condition can be attributed to several significant factors, including a low rate of early diagnosis, a high rate of postoperative recurrence, and a rapid progression following chemotherapy.^[1]^ The oncogenesis and growth of malignant tumors encompass various links, phases, and genes, which include initiation, progression, malignant conversion, invasion, and metastasis. The primary emphasis of traditional cancer research lies on investigating the intrinsic modifications occurring within tumor cells, encompassing both genetic and phenotypic changes. However, the continuous progress of gene and molecular biology technology has shown the complex mechanisms of the tumor microenvironment in tumor growth.^[2,3]^

In recent years, there has been a gradual deepening of study on the tumor microenvironment (TME). TME is a multifaceted and intricate milieu that provides an optimal setting for the proliferation of tumor cells. It mostly consists of stromal cells, immune cells, and the extracellular matrix. The transcriptional machinery of epigenetics plays a pivotal role in various aspects of tumor biology, including tumor epigenetics, tumor formation, evasion of immune response, and the process of metastatic invasion. The involvement of immune cells and the host immunological response are crucial components of the tumor microenvironment, frequently associated with tumor progression.^[4]^ Based on relevant studies, immunological variables have been identified as reliable and independent prognostic markers, surpassing the tumor, node, metastasis (TNM) stage in terms of accuracy.^[5]^ According to a recent study, immunotherapy is a significant component of anti-tumor treatments, encompassing active vaccination, adoptive cell transfer therapy, and immune checkpoint suppression. A number of medications are currently being assessment in clinical trials, and the results have demonstrated a distinct therapeutic application.^[6]^ Tumor infiltrating lymphocytes (TILs) play a significant role in the TME, with cluster of differentiation 3 (CD3^+^), CD4^+^, and CD8^+^ T cells being the predominant subgroups of TILs. Regulatory T cells (Tregs) are a distinct population of CD4^+^ T cells that possess the crucial role of suppressing T cell-mediated immune responses. The FoxP3^+^ transcription factor plays a crucial role in the induction of immunosuppressive activity and serves as a distinctive surface identifier for Tregs.^[7]^

Prior research has demonstrated a negative correlation between the presence of tumor infiltrating FoxP3^+^Tregs and the prognosis of the majority of individuals afflicted with malignant tumors.^[8]^ However, the predictive significance of this factor in biliary tract cancer remains a subject of controversy. Hence, the objective of this meta-analysis is to assess the association between tumor infiltrating FoxP3^+^Tregs and the prognostic outcomes of individuals diagnosed with biliary tract cancer.

## 2. Materials and methods

The systematic review was conducted, and the results were presented in adherence to the guidelines outlined by the Preferred Reporting Items for Systematic Reviews and Meta-Analyses.^[9]^ Ethical approval and informed consent were not deemed necessary for this work as the data utilized were solely obtained from published literature. The process of conducting a literature search, determining eligibility criteria, extracting data, and assessing study quality was carried out by 2 investigators in a manner that ensured independence. Any potential conflicts were effectively resolved through a process of constructive dialogue, ultimately leading the 2 researchers to reach a mutually agreeable compromise.

### 2.1. Search strategy

On January 6, 2023, a comprehensive search was conducted across 4 electronic databases, namely PubMed, Embase, Web of Science, and Cochrane Library. The search was not restricted by any time limitations. The vocabulary and syntax were deliberately modified in accordance with the database. The specific search terms of PubMed were: (“biliary tract cancer” OR “gallbladder cancer” OR “cholangiocarcinoma” OR “extrahepatic cholangiocarcinoma” OR “intrahepatic cholangiocarcinoma”) AND (“tumor infiltrating lymphocytes” OR “regulatory T cell” OR “FoxP3+”) AND (“prognosis” OR “predict” OR “impact” OR “detect” OR “risk” OR “overall survival” OR “recurrence” OR “mortality” OR “outcome”). There were no restrictions imposed on the use of any particular language. Additionally, a manual screening of reference lists including relevant papers was conducted to identify any potential additional data.

### 2.2. Inclusion criteria

The studies incorporated in the systematic review were required to satisfy the following criteria: The patients’ diagnosis of biliary tract cancer was confirmed through histopathological examination. The study detected the presence of FoxP3^+^ Tregs in the tumor tissue. The study reported on the association between FoxP3^+^ Tregs and overall survival time (OS). The literature presents relative risk (RR) along with its corresponding 95% confidence interval (95%CI) or provides adequate data for calculation. In cases where multiple studies involving the same population are published, preference is given to the study with the largest sample size or the most recently published study.

The exclusion criteria were defined as follows: There are several types of literature that may not meet the standards of academic rigor. These include: literature that has been published multiple times, documents that have incomplete or unclear analytical data and inconsistent result indicators, documents that exhibit low quality and lack original data, and case reports, commentaries, expert opinions, and narrative reviews.

### 2.3. Data extraction

The process of literature screening and data extraction will be conducted by 2 evaluators in a manner that ensures independence. The results obtained by each evaluator will be cross-checked, and in the event of any discrepancies, they will be thoroughly discussed and resolved through consensus. During the process of reviewing the literature, it is advisable to begin by examining the title and abstract. After eliminating any literature that is clearly irrelevant, it is then recommended to proceed with reading the whole text in order to ascertain its inclusion or exclusion. The necessary data was extracted and documented in standardized Excel files, which encompassed the last name of the primary author, the year of publication, the country of origin, demographic details of the participants (such as the number of patients, type of cancer, and stage of cancer), the testing methodology employed for FoxP3^+^ Tregs, the designated cutoff value, and the overall survival or other relevant summary effect measures.

### 2.4. Quality assessment

The assessment of the studies’ quality was conducted utilizing the New Castle-Ottawa Scale (NOS).^[10]^ The scale was partitioned into 3 distinct domains, namely selection, comparability, and outcome/exposure, each consisting of 9 items, resulting in a total of 9 points. Two reviewers evaluated the 9 NOS components as follows: the representativeness of the exposed cohort, the selection of the nonexposed cohort (selection bias), the ascertainment of exposure, the demonstration of outcome, the comparability of cohorts (comparability bias), the assessment of outcome, the length of follow-up, and the adequacy of follow-up of cohorts (outcome bias). The study quality was categorized into 3 levels, namely low, medium, and high, which were represented by scores ranging from 0 to 3, 4 to 6, and 7 to 9, respectively.

### 2.5. Statistical analyses

The variability among research was evaluated by employing chi-square statistics and further characterized by considering the magnitude of I^2^. The assessment of heterogeneity among the studies included in the analysis was conducted using the I^2^ statistic. A value of 0% for I^2^ suggests the absence of observed heterogeneity, while values exceeding 50% indicate the presence of significant heterogeneity. The relative risk in each article is standardized, followed by the utilization of random effects models for their aggregation. Furthermore, a sensitivity analysis was conducted to assess the impact of individual studies on the overall pooled results when employing the Leave-One-Out strategy. The present study investigated the presence of publication bias in meta-analyses comprising a minimum of 10 eligible publications, utilizing the symmetry of the funnel plot and conducting Egger test. To assess the potential impact of publication bias on the estimated effects, hypothetical negative unpublished studies were imputed, and the resulting funnel plot was examined for asymmetry. In all statistical tests, a 2-sided *P* value <.05 was deemed to be statistically significant. The data analysis for this study utilized Stata version 17, developed by StataCorp in College Station, TX, USA. The software was employed to analyze data obtained from randomized controlled trials that met the specified inclusion criteria.

## 3. Results

### 3.1. Search results and study selection

**Figure 1.**
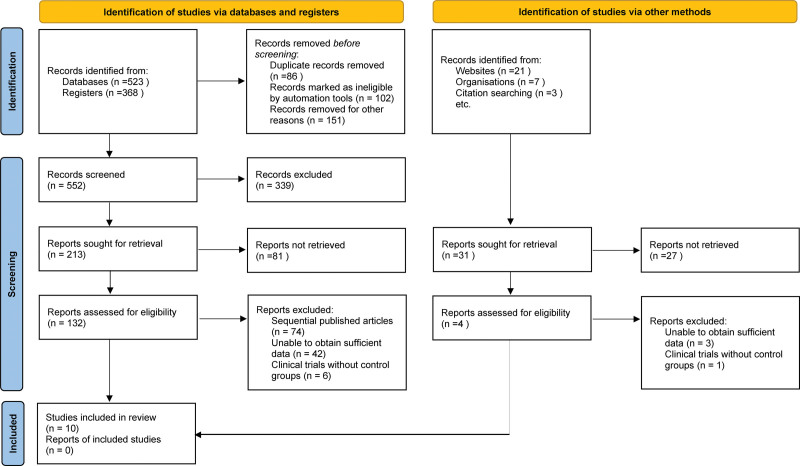
Selection process of included studies.

A total of 891 relevant literature sources were identified during the initial search of electronic databases. After eliminating redundant material, reviewing titles and abstracts, and applying rigorous inclusion and exclusion criteria, a total of 21 relevant articles were identified, while 11 were deemed ineligible for further analysis. In conclusion, a total of ten items were incorporated. Figure 1 displays the method and outcomes of the literature screening.

### 3.2. Study characteristics

The research incorporated in this analysis were published within the timeframe of 2013 to 2020. A comprehensive analysis encompassing 10 trials.^[11–20]^ incorporated a cohort of 1284 individuals diagnosed with biliary tract cancer. Tumor types encompass 3 distinct forms of biliary system cancer, namely intrahepatic cholangiocarcinoma, extrahepatic cholangiocarcinoma, and gallbladder carcinoma. There were 2 investigations that encompassed patients with 3 distinct subtypes of biliary tract cancer.^[12,13]^ Among these studies, Goeppert et al specifically examined the relationship between FoxP3^+^Tregs and OS within each subtype.^[12]^ Hence, these 3 distinct pieces of literature are considered as separate data sources. Table 1 presents the characteristics of the studies that were included in this systematic review.

**Table 1:** Characteristics of studies included in the meta-analysis.

Author	Year	Country	FoxP3^+^ Tregs (High/ Low)	Tumor type	TNM Stage	T cell detection method	T cell biomarker
Asahi	2020	Japan	69 (21/48)	ICC	II ~ IV	IHC	FoxP3 +
Wang	2020	China	238 (115/123)	GBC	I ~ IV	IHC	FoxP3 +
Kinoshita	2020	Japan	127 (64/63)	BTC	I ~ IV	IHC	FoxP3 +
Jing	2019	China	153 (NA)	ICC	I ~ IV	IHC	CD4 + FoxP3 +
Vigano	2019	Italy	53 (10/43)	ICC	NA	IHC	FoxP3 +
Fluxa	2018	Chile	77 (57/20)	GBC	I ~ IV	IHC	FoxP3 +
Kitano	2018	Japan	114 (85/29)	ECC	NA	IHC	FoxP3 +
Patil	2016	India	40 (17/23)	GBC	II ~ IV	FC	FoxP3 +
Goeppert	2013	Germany	309 (153/156)	BTC	I ~ IV	IHC	FoxP3 +
Zhang	2013	China	104 (52/52)	GBC	I ~ IV	IHC	FoxP3 +

FoxP3^+^ = Forkhead box P3, TNM = Tumor, node, metastasis.

### 3.3. Results of quality assessment

The NOS was utilized to evaluate the methodological quality of each randomized controlled trial. In a general sense, 3 research obtained a score of 8 points, while 7 studies achieved a score of 9 points. The trials did not employ blinding techniques, and there was a lack of evidence about allocation concealment. There were no discernible financial biases observed in any of the investigations. No trials were found to have missing outcome data, early stopping bias, or baseline imbalances. The summary of bias hazards and their related ratios is presented in Table 2.

**Table 2:** The quality assessment according to NOS of each cohort study.

study	Selection				Comparability	Outcome			Total score
	Representativ-eness of the exposed cohort	Selection of the non -exposed cohort	Ascertainment of exposure	Demonstration that outcome	Comparability of cohorts	Assessment of outcome	Was follow-up long enough	Adequacy of follow up of cohorts	
Asahi et al 2020	★	★	★	★	★★	★	★	★	9
Wang et al 2020		★	★	★	★★	★	★	★	8
Kinoshita et al 2020	★	★	★	★	★★	★	★	★	9
Jing et al 2019	★	★	★	★	★★	★		★	8
Vigano et al 2019	★	★	★	★	★★	★	★	★	9
Fluxa et al 2018	★	★	★	★	★	★	★	★	8
Kitano et al 2018	★	★	★	★	★★	★	★	★	9
Patil et al 2016	★	★	★	★	★★	★	★	★	9
Goeppert et al 2013	★	★	★	★	★★	★	★	★	9
Zhang et al 2013	★	★	★	★	★★	★	★	★	9

NOS = Newcastle-Ottawa Scale.

### 3.4. The results of meta-analysis

**Figure 2.**
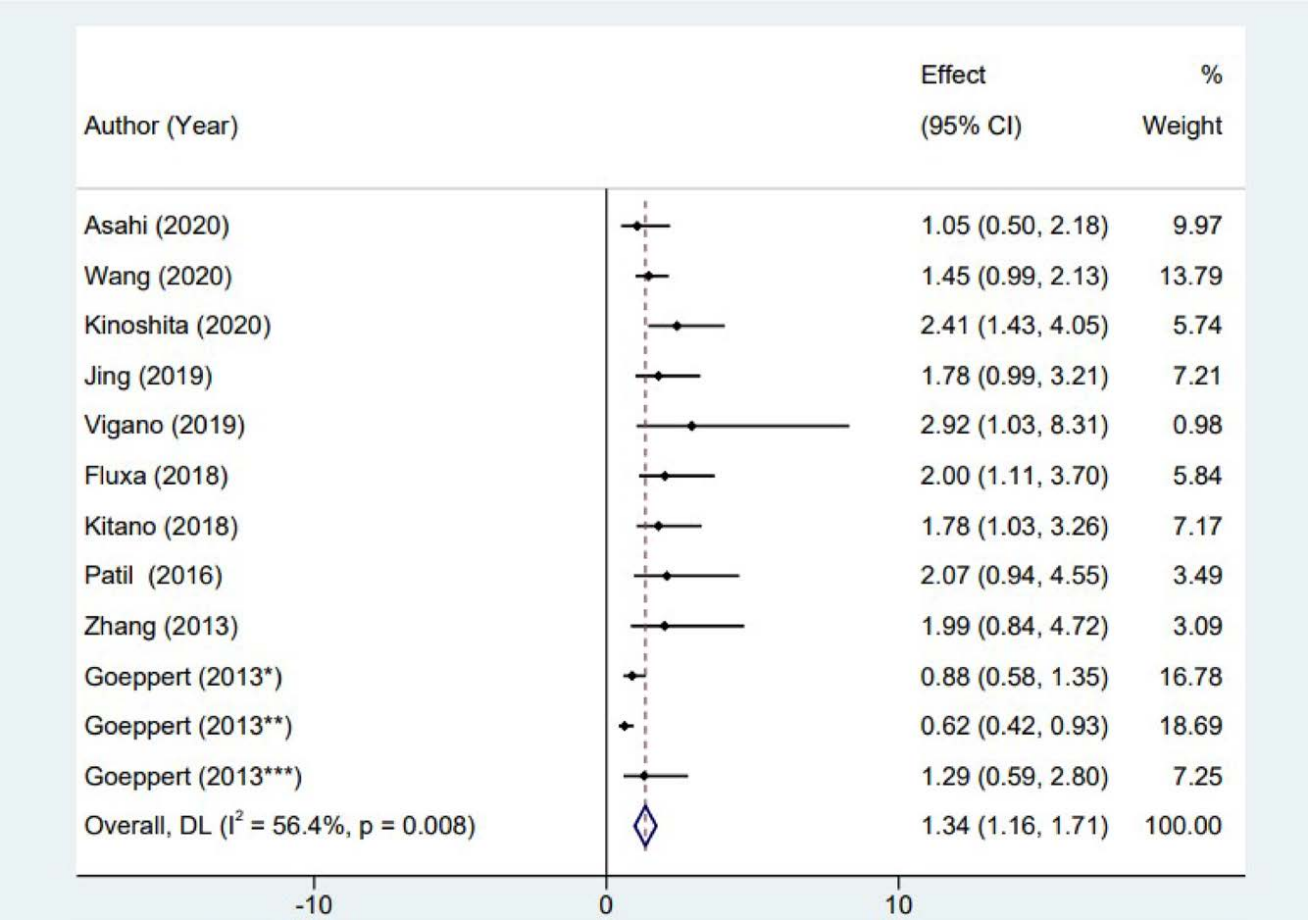
Forest plot of the correlation between regulatory T cells and the prognosis of biliary tract cancer.

The present study examines the correlation between tumor infiltrating FoxP3^+^ Tregs and OS in individuals diagnosed with biliary tract cancer. A comprehensive analysis was conducted, encompassing a total of 10 studies that met the inclusion criteria. The study revealed a degree of heterogeneity among the studies, as shown by an I^2^ value of 56.4% (*P* = .008). Consequently, the random effects model was chosen for the Meta-analysis. The findings of the study indicate a statistically significant reduction in OS among patients diagnosed with biliary tract cancer who exhibit a high presence of FoxP3^+^ Tregs (hazard ratio [HR] = 1.34, 95% confidence interval [CI] 1.16 to 1.71; *P* < .001; see Fig. 2).

### 3.5. The results of subgroup analysis

**Figure 3.**
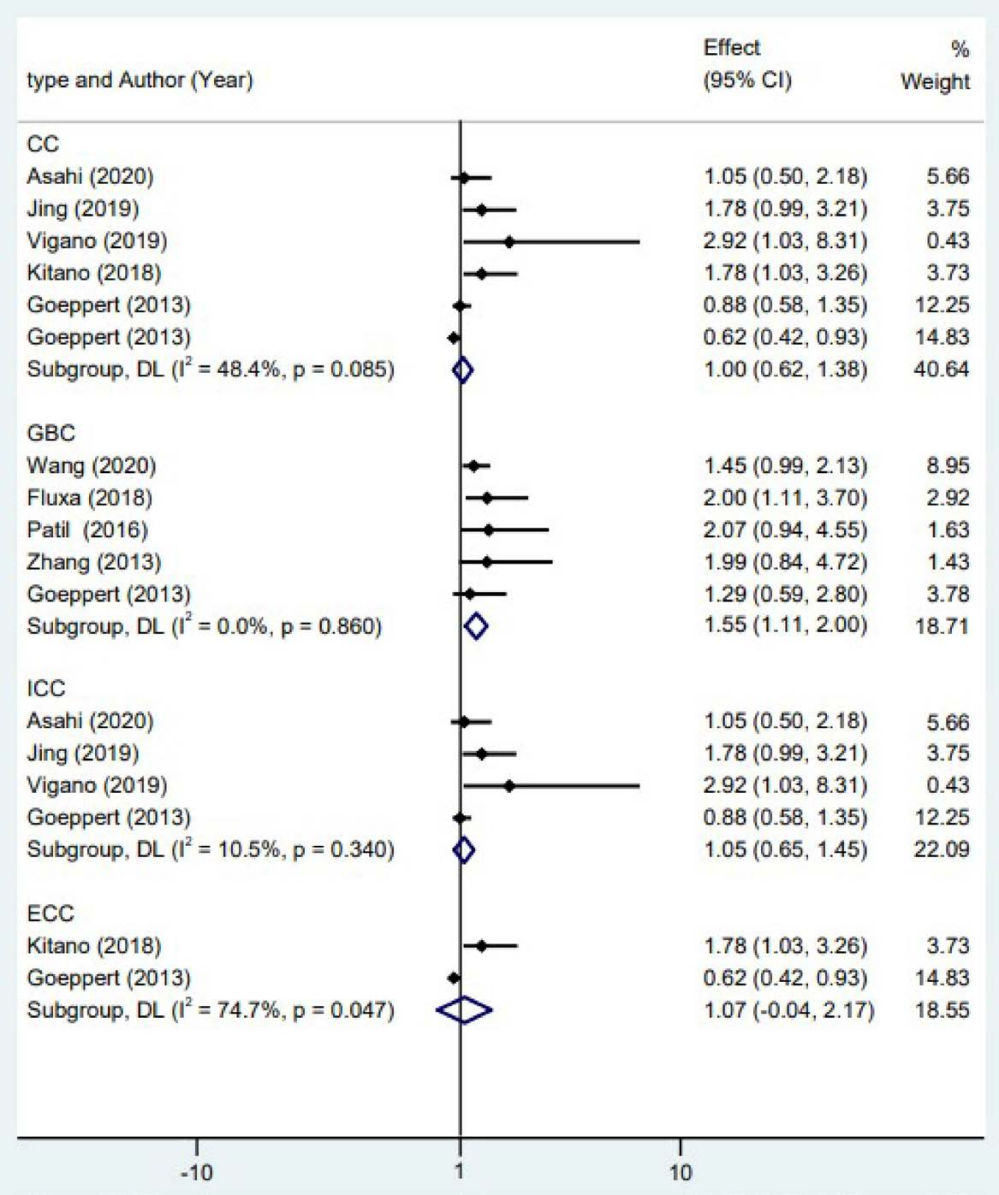
Forest plot of the correlation between regulatory T cells and the prognosis of different subtypes of biliary tract cancer. CC = cholangiocarcinoma, GBC = gallbladder cancer, ICC = intrahepatic cholangiocarcinoma, ECC = extrahepatic cholangiocarcinoma.

Biliary tract cancer refers to a malignancy that arises from several components of the biliary system and has notable heterogeneity. To assess the predictive significance of invasive FoxP3^+^ Tregs in patients with various subtypes of biliary tract cancer, a subgroup analysis was conducted specifically focusing on gallbladder carcinoma and cholangiocarcinoma (including intrahepatic cholangiocarcinoma and extrahepatic cholangiocarcinoma). The findings of the study indicated that the presence of FoxP3^+^ Tregs in patients with gallbladder cancer was associated with an unfavorable outcome (HR = 1.55, 95% CI 1.11 to 2.00; *P* < .001). There was no statistically significant association observed between OS and the presence of FoxP3^+^ Tregs in patients diagnosed with cholangiocarcinoma (HR = 1.00, 95% CI 0.62 to 1.38; *P* > .05). Similarly, no correlation was found between prognosis and FoxP3^+^ Tregs in patients with intrahepatic cholangiocarcinoma (HR = 1.05, 95% CI 0.65 to 1.45; *P* > .05) or extrahepatic cholangiocarcinoma (HR = 1.07, 95% CI -0.04 to 2.17; *P* > .05; see Fig. 3).

### 3.6. Publication bias

**Figure 4.**
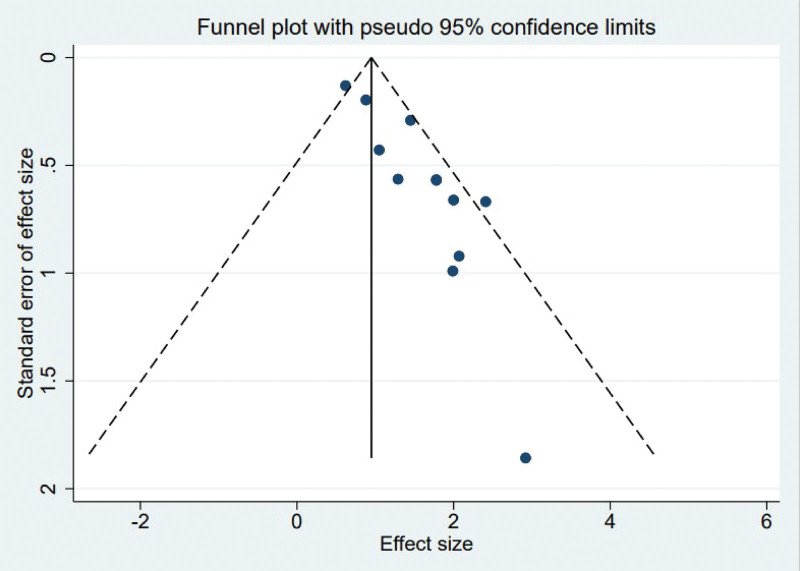
Funnel plots analysis for assessing publication bias.

The funnel plots generated using the observed study data exhibited symmetrical distribution, indicating the absence of substantial publication bias as seen by the absence of considerable asymmetry in the funnel plots (Fig. 4).

## 4. Discussion

Biliary tract cancer is a rapidly progressive tumor, and the current clinical treatment effect is not ideal. There is an urgent need for highly specific and sensitive biomarkers to evaluate the prognosis and help clinicians choose individual treatment strategies. However, at present, biliary tract cancer still depends on TNM staging to judge prognosis and lacks reliable molecular markers.^[21]^The prognostic value of tumor-infiltrating lymphocytes has attracted increasing attention in an increasing number of tumors, and other cells, except for FoxP3^+^Tregs, are worthy of further exploration. Combining a variety of immune cells or tumor-infiltrating lymphocytes to establish a prognostic model is the trend of future research, which may be an important supplement to the prognostic system of TNM staging.^[11,17]^ Prior to that, it is critical to comprehend the distribution characteristics and clinical association of tumor infiltrating lymphocytes in each specific tumor kind, along with the intricate interplay between them.^[4]^

Previous studies have demonstrated that Tregs possess immunosuppressive properties.^[6]^ Specifically, Tregs exert their immunosuppressive effects through 2 mechanisms. Firstly, they directly inhibit the cytotoxic activity of T cells by engaging in cell-to-cell contact. Secondly, Tregs suppress the function of effector T cells by releasing regulatory cytokines, including interleukin-10 (IL-10) and transforming growth factor-beta (TGF-β). The FoxP3^+^ protein plays a crucial role in the immunosuppressive function of Tregs. It achieves this by directly inhibiting the expression of the IL-2 gene and enhancing the transcription of the cytotoxic t-lymphocyte-associated protein 4 (CTLA4) gene. The association between immunosuppression and patients’ unfavorable prognosis has been observed. Currently, there is ongoing debate on the prognostic significance of FoxP3^+^Tregs in individuals diagnosed with digestive tract malignancies.^[22]^ FoxP3^+^Tregs have been found to have contrasting effects on various digestive system tumors, such as liver cancer and gastric cancer, where they are considered unfavorable prognostic factors.^[23]^ However, in the case of colorectal cancer, the results are contrary to this trend.^[24]^ This discrepancy may be attributed to the inhibitory effects of FoxP3^+^Tregs on inflammation and immune response, which are caused by the invasion of intestinal bacteria. Zhang et al^[14]^ highlighted that the FoxP3^+^Tregs possess both tumor immunity inhibitory capabilities and inflammatory regulatory functions, which can have favorable effects on the host. The overall impact of these functions is contingent upon the dominant role played by either mechanism in tumor formation. Furthermore, it has been observed via several studies that there is a correlation between elevated levels of FoxP3^+^ Tregs and increased levels of CD8^+^ T cells. This suggests that the favorable impact of FoxP3^+^ Tregs on prognosis may be attributed to the immunosuppressive negative feedback mechanism.^[25]^ The prognostic significance of FoxP3^+^ Tregs in individuals diagnosed with biliary tract cancer remains uncertain (Figs. 2 and 3). The findings of this study demonstrate a correlation between biliary tract cancer and reduced OS, which aligns with the immunosuppressive impact of the disease. It has been determined that FoxP3^+^ Tregs have the capacity to facilitate the development of an immunosuppressive milieu within biliary tumors, hence augmenting tumor cell proliferation, invasion, and metastasis, ultimately leading to an unfavorable prognosis.

Based on our subgroup analysis, it is evident that the association of FoxP3^+^ Tregs with prognosis significantly varies between gallbladder carcinoma and cholangiocarcinoma (Fig. 3). Although these 3 tumor subtypes all originate from bile duct epithelial cells, with the in-depth understanding of their molecular pathogenesis and the development of histopathology, more and more studies have shown that there is obvious genetic heterogeneity in different parts of biliary tract tumors.^[26]^ And for biliary tract tumors, T lymphocytes are a very complex and heterogeneous cell group, which can appear in different stages of tumorigenesis and development. The subgroup analysis highlighted a distinct prognostic relationship between tumor infiltrating lymphocytes in gallbladder carcinoma compared to cholangiocarcinoma, underscoring the potential differences in anti-tumor immunity and tumor microenvironment between these subtypes (Fig. 3). Secondly, another concern is CD8^+^T cells. Infiltration of CD8^+^T cells can reduce tumor cell metastasis and is associated with higher survival rate and lower risk of recurrence in patients with biliary tract cancer.^[19,27]^ High FoxP3^+^ Tregs/CD8^+^T cell value was found to be associated with poor OS and disease-free survival in lung adenocarcinoma, pancreatic cancer and other tumors.^[28,29]^ It can be seen that the increase of FoxP3^+^ Tregs infiltration and the decrease of CD8^+^T cell infiltration contribute to the formation of immunosuppressive TME and lead to poor prognosis. However, Goeppert et al^[12]^ found that the number of FoxP3^+^ Tregs infiltration was positively correlated with CD8^+^T cells. Therefore, there may be a negative feedback mechanism of FoxP3^+^Tregs immunosuppression in TME of biliary tract cancer, which weakens the immunosuppressive effect of FoxP3^+^Tregs to some extent. In a word, the result that FoxP3^+^Tregs has no effect on the prognosis of patients with cholangiocarcinoma may be the result of the intertumoral heterogeneity of gallbladder carcinoma and cholangiocarcinoma, the negative immune feedback mechanism of FoxP3^+^Tregs and anti-inflammation. More in-depth study of the mechanism is needed in the future. Of course, this conclusion may also be limited by the number of studies, so we should continue to pay attention to relevant clinical studies.

Our findings underscore the prognostic significance of FoxP3^+^ Tregs in biliary tract tumors, notably gallbladder cancer. Agents like anti-CD25 antibodies and denileukin diftitox show promise in depleting Tregs. For biliary tract cancers, combining Treg-targeted therapies with checkpoint inhibitors like anti-PD1/PDL1 may enhance antitumor effects by counteracting Treg suppression and amplifying effector T cell activity. Modifying the tumor microenvironment, possibly through CCL22 or CCR4 inhibition, may deter Treg recruitment. The varied prognostic implications of FoxP3^+^ Tregs in different biliary tract cancers suggest a tailored treatment approach. Rigorous preclinical testing and clinical trials are imperative to assess the viability of these strategies.

This study has several limitations. Some HR values were indirectly derived from Kaplan–Meier survival curves, introducing potential errors. While our results partly rely on univariate analyses, they might overlook confounding factors. The limited number of studies included emphasizes the need for continued research. We did not differentiate between intratumoral and peritumoral FoxP3^+^ Tregs, an important distinction for tumor immunity. Furthermore, despite rigorous article selection, meta-analyses are constrained by original data quality, limiting detailed insights on individual patient characteristics. Another limitation is our lack of a deep dive into the specific mechanisms driving differential FoxP3^+^Tregs expression between the cancer types. Nonetheless, our study provides pivotal, though broad, insights into the role of FoxP3^+^ Tregs in biliary tract cancer prognosis.

## 5. Conclusions

Our findings indicate that elevated levels of tumor-infiltrating FoxP3 + Tregs correlate with decreased OS in patients with gallbladder carcinoma, supporting their role as a prognostic marker in this subtype. However, such an association was not observed in cholangiocarcinoma, suggesting that the prognostic value of FoxP3 + Tregs may not be uniformly applicable across all biliary tract cancers.

## Author contributions

**Data curation:** Pengcheng Cai.

**Formal analysis:** Pengcheng Cai.

**Investigation:** Zhongli Wu.

**Methodology:** Xingjian Yang.

**Resources:** Pengcheng Cai, Xingjian Yang, Na Wang.

**Software:** Zhongli Wu, Na Wang.

**Supervision:** Yong Yang.

**Validation:** Yong Yang.

**Writing – original draft:** Pengcheng Cai.

**Writing – review & editing:** Yong Yang.
